# Coal seam in-situ inorganic analysis based on least angle regression and competitive adaptive reweighted sampling algorithm by XRF–visNIR fusion

**DOI:** 10.1038/s41598-022-27037-6

**Published:** 2022-12-26

**Authors:** Lei Zhu, Wenzhe Gu, Tianqi Song, Fengqi Qiu, Qingya Wang

**Affiliations:** 1 China Coal Energy Research Institute Co., Ltd., Xi’an, 710054 China; 2 School of Energy and Mining Engineering, China University of Mining and Technology (Beijing), Beijing, 10083 China; 3 State Key Laboratory of Nuclear Resources and Environment, East China University of Technology, Nanchang, 330013 China; 4 School of Automation Engineering, University of Electronic Science and Technology of China, Chengdu, 611731 Sichuan China

**Keywords:** Cheminformatics, Geochemistry, Fluorescence spectroscopy, Near-infrared spectroscopy

## Abstract

The fusion of X-ray fluorescence spectroscopy (XRF) and visible near infrared spectroscopy (visNIR) has been widely used in geological exploration. The outer product analysis (OPA) has a good effect in the fusion. The dimension of the spectral matrix obtained by OPA is large, and the Competitive Adaptive Reweighted Sampling (CARS) cannot cover the whole spectrum. As a result, the selected variables by the method are inconsistent each time. In this paper, a new feature variable screening method is proposed, which uses the Least Angle Regression (LAR) to select the high dimensional spectral matrix first, and then uses CARS to complete the secondary selection of the spectral matrix, forming the LAR-CARS algorithm. The purpose is to make the sampling method cover all the spectral data. XRF and visNIR tests were carried out on three cores in two boreholes, and a cross-validation set, validation set and a test set were established by combining the results of wavelength dispersion X-ray fluorescence spectrometer and ITRAX Core scanner in the laboratory. The quantitative model was established with the Extreme Gradient Boosting (XGBoost) and LAR-CARS was compared to these other algorithms (LAR, Successive Projections Algorithm, Monte Carlo uninformative variables elimination and CARS). The results showed that the RMSEP values of the models established by the LAR-CARS for six rock-forming elements (Si, Al, K, Ca, Fe, Ti) were relatively small, and the RPD ranges from 1.424 to 2.514. All these results show that the high-dimensional matrix formed by XRF and visNIR integration combined with LAR-CARS can be used for quantitative analysis of rock forming elements in in-situ coal seam cores, and the analysis results can be used as the basis for judging lithology. The research will provide necessary technical support for digital mine construction.

## Introduction

X-ray fluorescence (XRF) and near infrared spectroscopy technology (visNIR) are widely used in geological exploration, soil pollution investigation, inorganic and organic content analysis^1^. In recent years, the construction of digital mines has attracted a lot of attention, and the application of new technologies has promoted the digital process of core exploration^2^. In particular, XRF and visNIR have made great progress in the field of digital core. For example, portable single-optical path raster scanning NIR mineral analyzer developed by the Institute of Intelligent Instruments and Measurement and Control Technology of Jilin University, near-infrared mineral analyzer developed by Nanjing Institute of Geology and Mineral Resources, in-site portable XRF analyzer developed by Chengdu University of Technology^3^. The development of these small instruments provides technical support for digital mine construction.

XRF and visNIR are both rapid and nondestructive detection techniques, which have been used in digital mining for decades. However, due to the physical limitations of XRF and visNIR, many factors can affect the accuracy and reproducibility of in-situ analysis. Therefore, the spectral fusion of XRF and visNIR was proposed and also applied in the characterization of soil, minerals and element content^4^. Rios et al*.*^5^ introduced visNIR as a supplementary detection technology to make up for the deficiency of XRF in mineral characterization, and studied the internal characteristics of hematite and granite-greenstone ore in detail. Haavisto^6^ applied visNIR and XRF analyzer together in the content determination of floating slurry to obtain high-frequency detection results and sudden changes in grade. Wang et al*.*^7,8^ set up visNIR and XRF spectral fusion equipment to carry out quantitative analysis of four minerals in tungsten ore flotation process, and the results can be used for accurate ratio of flotation slurry. Marini et al*.*^9^ performed in-situ quantitative analysis of mineral samples in The Mediterranean Basin by fusion of NIR and XRF combined with stoichiometry and obtained good results. At the same time, the fusion devices of the two technologies have also been developed, such as ITRAX Core Scanner^10^, Avaatech Scanner^11^, DMT Core Scan^12^. In recent years, numerous studies have shown that the fusion of XRF and visNIR by outer product analysis (OPA) method can obtain richer characteristic spectra^13–15^, and the established quantitative model has better robustness.

Outer product analysis (OPA) is accomplished through the Kronecker product form of two detection signals^16^. However, the data volume after fusion is large, the dimension of variables is high, and there is interference of multicollinearity problem. When using partial least squares or support vector machines to build quantitative model of mineral or element, the prediction ability and robustness of the model will be reduced. It is particularly important to select appropriate variables for data with high dimensions. At present, more and more screening methods of characteristic variables have been proposed, such as variable selection method based on statistics^17^, variable selection method based on a single indicator and swarm intelligence optimization algorithm^18^. These algorithms provide a broad idea for variable selection.

The CARS uses Monte Carlo (MC) sampling and PLS regression coefficient as the index to select characteristic wavelength variables^19^. The adaptive reweighted sampling (ARS)^20^ as the core of the method to retain the wavelength points with heavy weight (large absolute value of regression coefficient) in the PLS model, and select the optimal variable subset combination according to the principle of minimum root mean square error of cross-validation. Many research results show that the CARS has simple structure, quick operation speed, but covering all OPA variables with MC sampling is very difficult. The number of extracted variables is big (more than 4,000,000 using XRF and visNIR data), the models are prone to overfitting, and have poor stability, so the improvement of CARS is a significant work^21^.

The Least Angle regression (LAR) combined with wavelength selection method of CARS (LAR-CARS) is proposed. A large number of characteristic variables are screened first and then refined again. This combination of methods can not only ensure the operation speed, but also ensure the MC sampling covers all the feature data. At present there is no report on the combination of inorganic element analysis method with it. In this study, core coal and naturally broken coal were collected in Zhashui mining area of China. XRF and visNIR were used to obtain the original spectral information of core. After necessary data pretreatment, model input was generated through OPA. The LAR-CARS algorithm was compared with the LAR, Successive Projections Algorithm (SPA), MC-UVE and CARS methods. XGBoost was used to establish a quantitative model and compare the statistical parameters of the model. The objective of this study was (a) evaluate and compare the correlation between the newly proposed LAR-CARS variable selection method and inorganic elements in coal seams and its importance for modeling performance, (b) explore the optimal modeling strategy for the analysis of inorganic elements in coal core by XRF and visNIR through exoproduct fusion, and evaluate the applicability of this method. The purpose of this study is to develop a data fusion technique that can be applied to in-situ core detection.

## Materials and methods

### Experimental area and sampling

**Figure 1:**
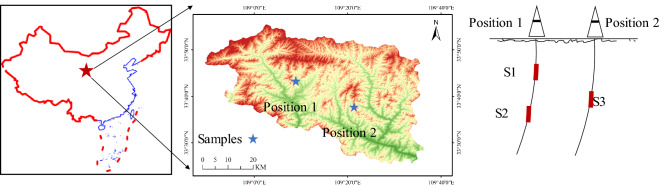
The location of the studied agricultural fields in Zhashui, China. (generated from publicly available geoscience data on the DataV.GeoAtlas platform; http://datav.aliyun.com/portal/school/atlas/area_generator, last access: 19 July 2022).

Drilling core sampling points are distributed in Zhashui County, Shaanxi province (108°49′48″–109°2′31″ E, 33°32′16″–33°41′13″ N), which is abundant in mineral resources, especially coal and iron resources. The sampling locations are shown in Fig. 1. S1 and S2 were taken out from Position 1, and S3 was taken out from Position 2. The cores of the three sections were not connected, they were all located in the coal reservoir, and the mineral types were similar. Three separate drill cores were studied here. Each core was 100 mm in diameter.

### Spectral collection

After core S1 and S2 were extracted, spectral tests were carried out. Olympus Delta Premium (DP-6000-C) Field-portable XRF Analyser; SDD detector; Rh target, 125 eV energy resolution, Amptek. Test parameters were: tube voltage 35 kV, current 40 μA, Test time 30 s. VisNIR Spectroscopy was recorded using a FieldSpec Pro FR visNIR Spectrometer (PANalytical Inc., formerly Analytical Spectral Devices-ASD, Boulder, CO). The spectral range was 350–2500 nm. The sampling interval was 3 nm (350–1000 nm) and 10 nm (1000–2500 nm), and the sampling resolution of the spectra was 1 nm. A Spectralon^®^ panel with 99% reflectance was used to calibrate the spectrometer and each sample was randomly scanned 10 times. The average of three results with the best signal-to-noise ratio were the spectrum of the samples.

The XRF and visNIR tests were conducted at 50 mm intervals, assisted by a marker length tape parallel to the core axis. Fractures were avoided when testing, requiring that each test be directly against solid rock or coal. Move the measuring point slightly if necessary and mark the test point finally. 318 points with 656 spectral data (XRF: 322, NIR: 334) were detected for analysis by S1 and S2. 295 points (XRF: 295, NIR: 295) were left after kicking out operational errors and obvious differences caused by changes in experimental environment. 174 spectral data (XRF: 87, NIR: 87) were collected from S3 and no abnormal data was found after inspection.

### Laboratory physicochemical analysis

4 g samples were taken from S1 and S2 according to the test points, dried at 105 °C for 1 h and cooled to room temperature. The samples were placed in a tablet press (Shanghai Shengli SL201 semi-automatic press machine). High-density polyethylene dry powder was used as the substrate, and 0.4 g maltodextrin powder was added to the samples that were difficult to form, kept at 40 T pressure for 20 s. Pressed into a diameter of 32 mm, outer diameter of 40 mm wafer, a total of 295 samples. Wavelength dispersive X-ray fluorescence spectrometer (Axios Pw4400, Panaco, the Netherlands) was used during this research, and the test parameters are shown in Table 1.

**Table 1:** Test conditions of wavelength dispersive X-ray fluorescence analyzer.

Channel	Spectral lines	Crystal	Collimator (μm)	The detector	Tube voltage (kV)	Tube current (mA)
Si	K_α_	Li200	150	Scient	60	60
Al	K_α_	Li200	150	Flow	60	60
K	K_α_	Li200	150	Scient	60	60
Ca	K_α_	Li200	150	Flow	60	60
Fe	K_α_	Li200	150	Scient	60	60
Ti	K_α_	Li200	150	Scient	60	60
S	K_α_	Li200	150	Scient	60	60

S3 was scanned and analyzed using ITRAX Core Scanner. Before scanning, the core scanner was corrected with the sample discretization results of WD-XRF to ensure the consistency of the instrument results. The sampling interval of instrument was 1 cm on the core, and the other parameters were set according to the Ref.^22^ to obtain the oxide content results, which were converted to the element content.

### Spectral pretreatment and out product fusion

XRF is characteristic spectrum. In the process of spectral acquisition, there will be a lot of noise information interference such as high-frequency random noise, baseline drift and scattering, which will affect the correlation between XRF spectrum and element content, and ultimately affect the reliability and stability of model establishment, so it is necessary to preprocess spectral data.

**Figure 2:**
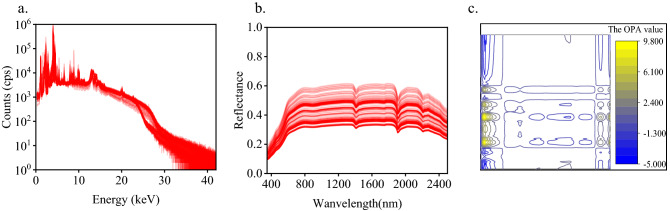
The acquired spectra, (**a**) XRF, (**b**) visNIR, (**c**) OPA.

Considering the large amount of noise at both ends of the detection range, the introduction of these noises will affect the performance of the model. First, delete the data at both ends and keep the data in the middle which contain more information about the element content. The visNIR reflection spectrum intercepted 450–2450 nm (2000 channels) and converted the reflection spectrum into an absorption spectrum. The XRF spectrum was selected from 0.405 to 42.105 keV (2000 channels). The purpose was to eliminate the influence of noise and low-energy radiation. The sym4 wavelet function was used for wavelet transform denoising and the adaptive iterative weighted penalty least squares (airPLS) method was used to calibrate the baseline^23^. Then, baseline correction was performed on both spectra. Savitzky–Golay (SG) smoothing^24^ with 15 window size and second order polynomial, Standard Normal Variate (SNV)^25^ were used to preprocess the raw data. At this time, the numerical range of visNIR spectrum was − 3.92 to 4.59, and the numerical range of XRF spectrum was − 1.56 to 3.37, which solved the dimensional inconsistency between different physical quantities. This problem is commonly encountered in data fusion. The pre-processed spectral data are shown in Fig. 2a,b. Because 370 spectral data are displayed, the transparency of each data is set to 90%. The darker the color, the more concentrated the location of the spectrum appears.

The OPA is calculated by the Kronecker product of XRF and visNIR, and the fusion process is detailed in Ref.^26^. XRF and visNIR spectra each have 2000 channels. After fusion, the spectral variable reaches 2000 × 2000. The fused matrix is shown in Fig. 2c. The spectrum pretreatment and OPA steps are completed.

### The least angle regression and competitive adaptive reweighting sampling algorithm (LAR-CARS)

#### Algorithm principle

LAR-CARS consists of LAR and CARS. LAR is first used for the matrix with higher dimensions to complete the coarse selection of feature variables, and then CARS is used to complete the fine selection of feature variables. Least Angle Regression algorithm (LAR)^27^ is a new machine algorithm proposed based on linear regression principle. The algorithm is faster than other methods in selecting characteristic variables. The principle of the algorithm is relatively simple. First, the first-order penalty function is constructed to make the coefficient of the variable zero, and then some invalid variables are removed to reduce the scale of the model and a model with higher explanatory degree is obtained. The linear regression model is shown as follows:

1 $$\min S(\overline{\delta }) = \parallel {\mathbf{y}} - \overline{\varvec{\mu }}\parallel^{2} = \mathop \sum \limits_{i = 1}^{n} \left( {y_{i} - \overline{\mu }_{i} } \right)^{2} = \mathop \sum \limits_{i = 1}^{n} \left( {y_{i} - \mathop \sum \limits_{j = 1}^{p} x_{ij} \delta_{j} } \right)^{2}$$

2 $$subject\; to\; { }\sum\limits_{j = 1}^{p} {\left| {\delta_{j} } \right|} \le t\quad \left( {t \ge 0} \right)$$

where $\left( {x_{i1} ,\;x_{i2} , \ldots ,\;x_{ip} } \right)$ is the independent variable corresponding to the ith sample, *y*_*i*_ is the dependent variable, and $\delta_{j}$ represents the regression coefficient of the *j*-th independent variable, and its constraint value is *t*. Under the constraint of formula (2), LAR algorithm adjusts $\delta_{i}$ to minimize the sum of the square variances of *y*_*i*_ and the regression variable $\overline{\mu }_{i}$. When the initial value of *t* is small, the coefficient of the independent variable $\delta_{i}$ with low variable dependence is directly set to zero, and these characteristic variables are eliminated. The remaining variables are reconstituted into sparse feature matrix. When *t* increases to a large value or a preset value, the constraint disappears, and the least square method is used to solve the data subset. The main method for determining *t* value is Akaikes Information Criterion (AIC)^28^.

**Figure 3:**
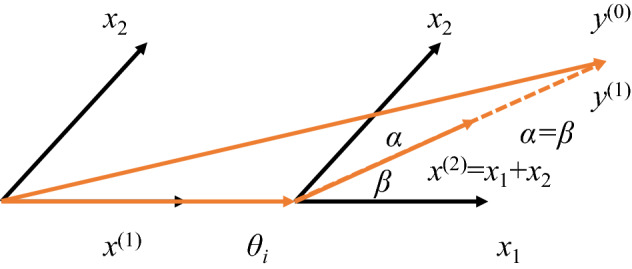
The two-dimensional diagram of LAR algorithm.

Concrete implementation process is (1) vector X variable coefficient of the initial value is set to 0, and then in the direction of feature vector *x*_*i*,_ find out the *y*^(0)^ that has the greatest correlation with the initial target residuals. (2) move the *x*_*i*_ by $\theta_{j}$ step length to *x*_*j*_, make the characteristic of residual vector ${\mathbf{y}}^{(0)} - \theta_{i} x_{i}$ and *x*_*j*_ have the same relevance (i.e., is located in the angle of the *x*_*i*_ and *x*_*j*_ just divide the line). (3) move along the angular bisector until the next variable *x*_*l*_ with the same residual correlation with (*x*_*i*_, *x*_*j*_) is found, and the cycle continues until all variables are selected. The two-dimensional plane calculation process of the algorithm is shown in Fig. 3.

CARS algorithm combines Monte Carlo sampling with PLS and establishes feature wavelength selection method based on the "survival of the fittest" rule in biological evolution^29^. Adaptive Reweighted Sampling (ARS) technology^20^ is used to screen out characteristic wavelengths with large absolute values of regression coefficients and remove those with small absolute values, so as to obtain a series of optimal subsets of variables. Then, the cross-validation method is used to determine the subset with the minimum RMSECV value in the variable quantum set, which is the optimal wavelength combination of the measured elements.

#### Algorithm steps

First, LAR was used for preliminary screening of the fused spectral feature variables, and CARS was used for further screening of the selected variables, so as to find the minimum set of feature energy points with excellent modeling performance and interpretation. The specific steps are as follows:

1. Assuming that the fused spectral data is an *i* × *l* matrix, the input model is used to solve the regression coefficient, and the regression variable matrix is constructed. The model solution is based on the principle of AIC minimization. The correlation of characteristic variable I is represented by 0 and 1, with 0 representing low correlation and 1 representing high correlation.
2. LAR model uses variable matrix to solve spectral characteristic variables:
  3 $${\overline{\mathbf{X}}} = {\mathbf{X}}\beta$$
  where ${\overline{\mathbf{X}}}$ is the characteristic variables selected by LAR model. **X** is the fusion spectral matrix by OPA and $\beta = \left[ {\beta_{1} ,\;\beta_{2} , \ldots ,\;\beta_{l} } \right]^{T}$ is the regression variable matrix.
3. The variables screened by LAR were randomly sampled 50 times by Monte Carlo sampling method. The regression analysis model is established by subset variables ${\overline{\mathbf{X}}}$ and target element quantity **Y**,
  4 $${\mathbf{Y}} = \overline{{\mathbf{X}}}_{i} {{\varvec{\upbeta}}} + {{\varvec{\upvarepsilon}}} = \overline{{\mathbf{X}}} \beta_{i} + {{\varvec{\upvarepsilon}}}$$
  The contribution matrix *β*_*i*_ can be expressed as
  5 $$\beta_{i} = \overline{{\mathbf{X}}}_{i}^{ - 1} ({\mathbf{Y}} - \varepsilon )$$
  The corresponding weight *w*_*i*_ of each feature variable is solved through the matrix
  6 $$\omega_{i} = \frac{{\left| {\overline{{\mathbf{X}}}_{i}^{ - 1} ({\mathbf{Y}} - \varepsilon )} \right|}}{{\sum\limits_{i = 1}^{p} {\left| {\overline{{\mathbf{X}}}_{i}^{ - 1} ({\mathbf{Y}} - \varepsilon )} \right|} }}\quad i = 1,\;2, \ldots ,\;p$$
4. Exponential decay functions were used to forcibly remove wavelength points with relatively small weights.
5. Wavelength with large absolute value of regression coefficient in PLS model was screened by N times adaptive reweighting sampling technology. RMSECV value of new variable subset generated each time was calculated and compared, and the variable subset with the smallest value was regarded as the optimal variable subset.

Finally, Matlab R2019b and Origin 2018.0 were used to complete the algorithm writing and chart drawing of LAR, SPA, MC-UVE and CARS. Firstly, the LOOCV set samples were modeled, and the single sample test and the fused OPA spectrum (2000 × 2000) were selected as the objects. LAR, SPA, MC-UVE, CARS and LAR-CARS algorithms were used for screening. The selection method of CARS variable was based on Monte Carlo cross validation, the maximum number of principal components was 50, the number of Monte Carlo operation samples was 50, the number of interactive verification samples was 10, and the number of operation times was 50. SPA variable selection method used vector projection analysis principle. Specific principles and steps of SPA and MC-UVE variable selection methods can be found in literature^30,31^ The computer's environment was configured as Intel Core i7-9750H @2.60 GHz, 16 GB (2667 MHz), Intel UHD Graphics 630 128 MB.

### Data analysis and evaluation indicators

Gradient boosting decision tree (GBDT) is an integrated learning algorithm based on decision tree. The base learner of GBDT adopts regression tree, and each tree fits the negative gradient of the loss function on the previous tree. Finally, the linear weighted sum of the results of all regression trees is taken as the output model. XGBoost is an efficient implementation of GBDT, and its base learner includes classification tree and regression tree. The analysis of coal seam inorganic elements in this study belongs to regression problem, so the base learner used in this study is regression tree. Compared with GBDT algorithm, XGBoost algorithm explicitly adds regularization terms in the objective function. When updating the base learner, GBDT generates the base learner iteratively according to the first derivative, while XGBoost updates the base learner not only according to the first derivative but also according to the second derivative. In addition, XGBoost algorithm also makes a lot of optimization during implementation. An XGBoost model with *n* trees can be expressed as:

7 $$y_{i} = \sum\limits_{n = 1}^{N} {f_{n} } \left( {x_{i} } \right)$$

where *y*_*i*_ represents the element content of the *i*-th sample, *x*_*i*_ represents the characteristic variable after spectral screening of the *i*-th sample, and *f*_*n*_ represents the prediction function of the *n*-th decision tree.

Gradient Boosting strategy is adopted for XGBoost model, which updates the model by updating the negative Gradient direction of the loss function, and its optimization model can be expressed as

8 $$\Omega (f) = \gamma T + \frac{1}{2} \parallel \lambda \omega\parallel ^{2}$$

9 $$O(\phi ) = \sum\limits_{i} l \left( {y_{i} ,y_{i} } \right) + \sum\limits_{n} \Omega \left( {f_{n} } \right)$$

where *l* (*y*_*i*_,*y*_i_) represents the loss function, i.e., the mean square error, Ω(*f*_*n*_) represents the regularization term, *γ* represents the model complexity, *T* represents the number of leaf nodes in the model, $\lambda$ represents the fixed coefficient, and $\omega$ represents the quantization weight vector of leaf nodes. On the basis of preserving the well-trained tree model, XGBoost model continuously substituted the derivative of the prediction function according to the loss function into the prediction function of the last round to update the prediction function, and finally obtained the prediction result through iterative calculation.

In order to comprehensively evaluate the performance of all models, Cores S1 and S2 were modeled and validated. Applicability analysis was performed with S3 cores. The modeling process was as follows: WD-XRF test samples (295) were used as cross-validation, and separate cores (75 points) were used to independently verify the model. Leave-One-Out-Cross-Validation (LOOCV) was used to train the model. In this way, one sample in the group of built models is eliminated, and the remaining samples are used to train the model to predict the composition of excluded samples, calculate the difference between the predicted value and the actual value, and repeat the process until all samples are removed once. The total error obtained is the prediction accuracy of the cross-validation root mean square Error (RMSECV) judgment model. The statistical distribution of LOOCV set and prediction set is shown in Table 2.

**Table 2:** Statistical characteristics of metal content.

Set type	Core	Metal	Max. (%)	Min. (%)	Mean (%)	SD (%)	CV (%)	Skewness	Kurtosis
LOOCV set(train set)	S1	Si	46.877	2.602	25.763	9.755	0.379	− 0.077	− 0.692
		Al	21.918	3.249	12.399	4.164	0.336	0.048	− 0.781
		K	3.441	0.288	1.784	0.718	0.403	− 0.004	− 0.812
		Ca	10.427	0.184	5.370	3.012	0.561	− 0.075	− 1.313
		Fe	11.534	0.355	5.835	2.953	0.506	0.127	− 1.121
		Ti	1.457	0.061	0.749	0.341	0.454	0.069	− 0.936
Validation set	S2	Si	43.270	3.474	21.847	9.883	0.452	0.238	− 0.793
		Al	22.615	3.132	10.664	4.597	0.431	0.393	− 0.459
		K	4.501	0.620	1.960	0.768	0.392	0.638	0.474
		Ca	12.099	0.393	4.976	2.897	0.582	0.375	− 0.608
		Fe	13.504	0.624	5.119	2.842	0.555	0.517	− 0.294
		Ti	2.565	0.150	1.099	0.474	0.431	0.691	0.333
Test set	S3	Si	36.738	9.009	21.655	6.126	0.283	0.361	− 0.404
		Al	17.809	3.133	10.706	2.715	0.254	0.350	0.535
		K	3.366	0.828	1.960	0.451	0.230	0.239	0.853
		Ca	10.674	1.792	5.094	1.678	0.329	0.220	0.410
		Fe	8.652	1.053	5.138	1.587	0.309	− 0.192	− 0.490
		Ti	2.022	0.541	1.078	0.297	0.276	0.229	0.121

**Figure 4:**
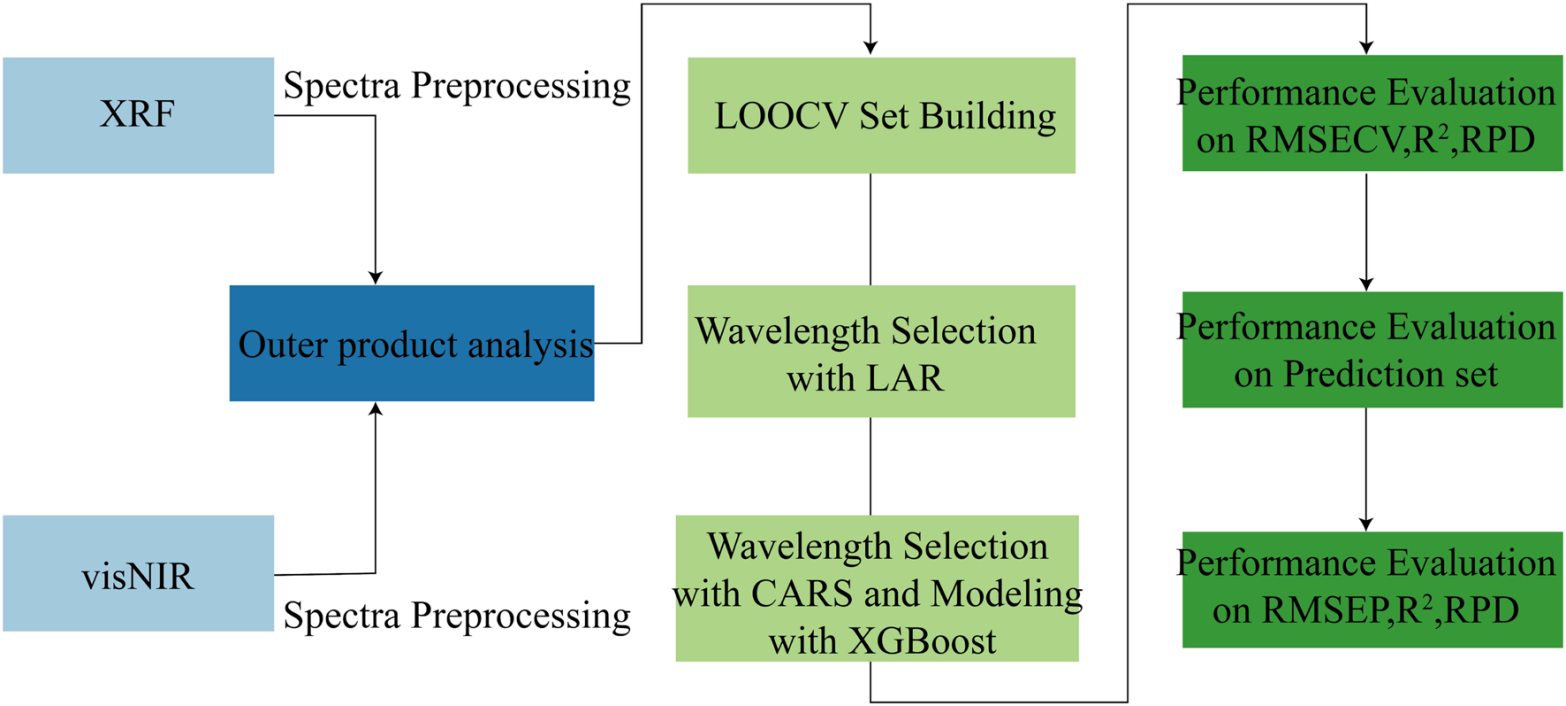
The Flow chart of proposed variable selection method by LAR-CARS.

The root mean square error of prediction (RMSEP) was obtained by predicting the single core mining point. The overall prediction accuracy of the model was measured by four parameters: coefficient of determination (*R*^2^), root mean square error of cross validation (RMSECV), RMSEP and relative deviation percentage (RPD). *R*^2^ reflects the stability of model establishment and validation. RMSECV and RMSEP are used to test the predictive power of the model^32^. RPD is the ratio of sample standard deviation to RMSE, which measures the predictive power of the model. When RPD < 1.4, this model cannot be used to predict samples. When 1.4 ≤ RPD < 2, the model is considered just good enough to be used for rough evaluation of samples. When RPD ≥ 2, the model has good predictive ability^33^. The LAR and CARS information processing flow chart presented in this paper is shown in Fig. 4. The parameters of the LAR-CARS algorithm were set as follows: the number of repetitions is 2048, the maximum number of latent variables of cross-validation is 10, the preprocessing method is center-centered, and the number of Monte card sampling is 50.

## Results and analysis

### Selection of spectral characteristic variables

**Figure 5:**
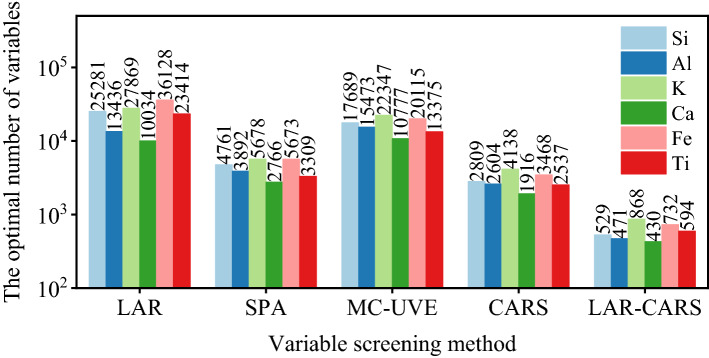
Number of optimal variables to filter.

Screening results are shown in Fig. 5. According to the results, the number of variables roughly selected by LAR is larger than other algorithms. According to the algorithm principle, the LAR principle is simple and many variables are screened out. CARS showed a small number of variables obtained from the four commonly used variable screening methods, with the number of variables ranging from 2537 to 4138. Compared with MC-UVE, SPA algorithm had a smaller number of variables, ranging from 2766 to 5678. MC-UVE had a large number of variables, ranging from 10,777 to 22,347. The results obtained are similar to previous reports^34^.

**Figure 6:**
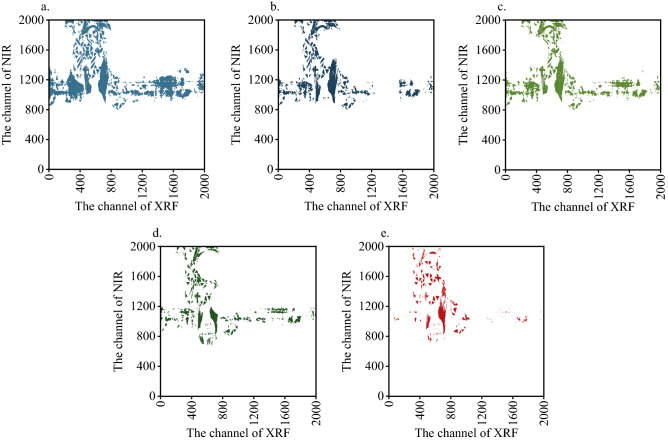
The feature selection of Ca OPA spectral distribution, (**a**) LAR, (**b**) SPA, (**c**) MC-UVE, (**d**) CARS, (**e**) LAR-CARS.

The number of screening variables directly affects the calculation speed of the model. The distribution of single element screening variables will be studied below. Figure 6 shows the distribution positions of the characteristic variables of calcium elements in the whole spectrum based on different extraction methods. As can be seen from the figure, the visNIR features extracted by LAR were mainly concentrated in 800–1200 channel, and the XRF features were mainly concentrated in 400–800 channel and about 1600 channel. The wavelength range of SPA screening was similar to that of LAR. Compared with LAR, SPA method reduced the visNIR features of 1300–1800 channel and XRF features of 1200–1600 channel. The LAR-CARS selected the lowest number of features, and compared with LAR results, the visNIR 1200–2000 channel density was diluted and the XRF 1000–2000 channel count was reduced. The results of LAR-CARS were concentrated around 800–1200 and 1600 channel in visNIR and 746 channel in XRF. The spectrum range of 800–1200 channel was 1149–1549 nm, represents the out-of-plane deformation vibration and C–H stretching vibration according the visNIR spectrometer handbook. These peaks had strong characteristics, but more interference. The 1600 channel corresponds to 1949 nm, where the stretching vibration of C–H double bond was mainly present. According to the XRF calibration results of pure elements, the 400–800 channel corresponds to 8.381–16.783 keV, which mainly correspond to the K_α_ characteristic peaks of Cu, Ni, Zn, Ga, As and other heavy metals.

There are K_α_ characteristic peak of Rh, Pd, Ag, Cd elements around the 1600 channel at 23.587 keV. LAR algorithm can filter out the Ca element characteristic peak 3.69 keV, while other algorithm do not selected the characteristic peak. The reason was that there are many disturbances near the Ca characteristic peak, such as K and Ar in the air, which can interference of Ca. The linear relationship between Ca peak intensity and content was severely affected, so the linear relationship is not strong, which is also the reason why Ca cannot be quantified by traditional XRF calibration method, which is similar to what was reported in literature^35^.

### Evaluation of feature selection

**Figure 7:**
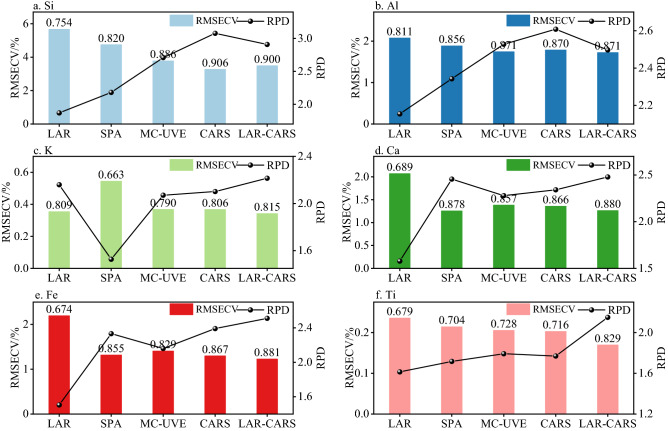
XGBoost modeling results of LOOCV set, (**a**) Si, (**b**) Al, (**c**) K, (**d**) Ca, (**e**) Fe, (**f**) Ti. *The bar chart is labeled *R*^2^ for this model.

In this section, the LAR-CARS algorithm was used to screen the wavelength variables of the sample spectrum, and compared with the LAR, SPA, MC-UVE and CARS algorithms, the six elements of Si, Al, K, Ca, Fe and Ti in the core, LAR-XGBoost, SPA-XGBoost, MC-UVE-XGBoost and CARS-XGBoost models were established respectively, and the process of model establishment was carried out according to Ref.^36^. The prediction effect of the established model was evaluated. The modeling effects of each model are shown in Fig. 7. In the quantitative model of Si, The *R*^2^ of LAR-XGBoost was the smallest, at 0.75, corresponding to the RMSECV value of 5.67%. The results of CARS-XGBoost and LAR-CARS-XGBoost were similar, where the RMSECV of CARS-XGBoost and LAR-CARS-XGBoost were 3.281% and 3.491%, respectively. The RPD values of the two models were 3.08 and 2.91. The two models achieved better prediction effect. It was worth noting that although MC-UVE screened a large number of feature wavelengths, up to 17,689, the prediction effect was improved compared with SPA, which was similar to what was reported in literature^31^. In the quantitative models of Al, the differences among the five models were not large, with *R*^2^ ranging from 0.811 to 0.871 and RMSECV ranging from 1.721 to 2.069%, with a range of 0.348%. The best model was CARS-XGBoost with *R*^2^ of 0.870 and RPD of 2.608.

In the quantitative model of K, SPA-XGBoost performed poorly, with *R*^2^ of 0.663. The prediction effects of the other four models were relatively close, with *R*^2^ range of 0.806–0.815. The best-performing model was LAR-CARS with an RMSECV of 0.334, which was 37% lower than the RMSECV 0.532 of the highest SPA-XGBoost model. Among the Ca quantitative models, the worst model was LAR-XGBoost, with *R*^2^ of 0.689 and RPD of 1.615. The prediction performance of the other four models was relatively close, with *R*^2^ ranging from 0.857 to 0.880. The best performing model was SPA-XGBoost with an RPD of 2.493, while the LAR-CARS was close to SPA-XGBoost with an RPD of 2.419. Among the quantitative models of Fe, the LAR-XGBoost model had the worst performance, with *R*^2^ of 0.674 and RMSECV of 2.166%. The other four models had similar prediction performance, with *R*^2^ ranging from 0.829 to 0.881. The model with the best performance was LAR-CARS-XGBoost and RPD was 2.599. Among the prediction models of Ti, the LAR-XGBoost model had poor prediction performance, with RPD of 1.553 and *R*^2^ of 0.679. The best performance model was LAR-CARS-XGBoost with RPD of 1.985 and RMSECV of 0.188%, which showed a significant improvement in performance compared with the other four models. In conclusion, the prediction effect of LAR-XGBoost was always poor in the six element prediction models, while the prediction effect of LAR-CARS and CARS was generally good.

### Effect evaluation of independent prediction sets

**Figure 8:**
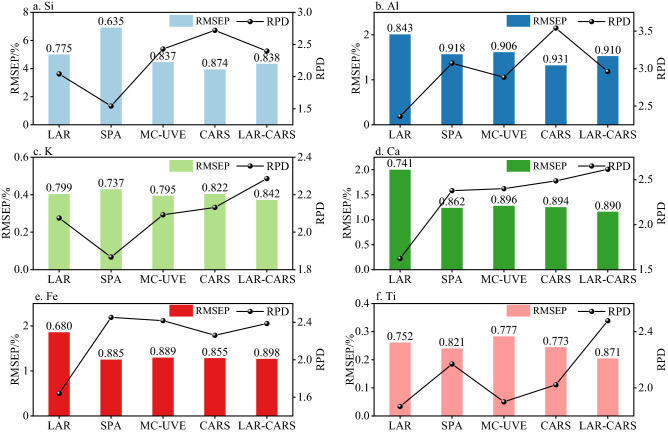
XGBoost modeling effect of validation set, (**a**) Si, (**b**) Al, (**c**) K, (**d**) Ca, (**e**) Fe, (**f**) Ti. *The bar chart is labeled *R*^2^ for this model.

XGBoost modeling was carried out according to the independent prediction set, and LAR, SPA, MC-UVE, CARS and LAR-CARS models were established for Si, Al, K, Ca, Fe and Ti, respectively. The prediction results are shown in Fig. 8. Among the Si prediction models, SPA-XGBoost had the worst prediction, with *R*^2^ of 0.635 and RMSEP of 6.903%. The CARS-XGBoost model had the best effect, with *R*^2^ of 0.874 and RPD of 3.930. LAR-CARS-XGBoost also performed well, with *R*^2^ of 0.838 and RMSEP of 4.324%. In the prediction model for Al, LAR performed worst, with *R*^2^ of 0.843 and RMSEP of 2.005%. The best performance was CARS-XGBoost with *R*^2^ of 0.931 and RMSEP of 1.316%. The LAR-CARS-XGBoost also had good performed, with *R*^2^ of 0.910 and RPD of 2.960. In the K prediction model, the performance of models was relatively balanced, the range of *R*^2^ was 0.737–0.842, and the range of RPD was 1.921–2.372. The best performing model was LAR-CARS-XGBoost, with *R*^2^ of 0.842, RPD of 2.372 and RMSEP of 0.338%. In the Ca prediction model, LAR-XGBoost had poor prediction performance, with *R*^2^ of 0.741. The best model was LAR-CARS-XGBoost, with *R*^2^ of 0.890 and RPD of 2.497. Among the Fe prediction models, LAR's prediction ability was poor, with *R*^2^ of 0.680. SPA, MC-UVE, CARS, and LAR-CARS showed similar performance. The *R*^2^ of the four models ranged from 0.855 to 0.898, and the RPD range was 2.282 to 2.523. In the prediction model of Ti, LAR-CARS-XGBoost had the best performance, with *R*^2^ of 0.871 and RPD of 2.583.

It can be seen from the above results that the feature variables screened by LAR have poor performance, while CARS and LAR-CARS screen fewer feature variables, and the established model had better stability and prediction performance. CARS had the best performance in Si and Al, and LAR-CARS had the best performance in K, Ca, Fe and Ti.

### The application of core in-situ inorganic analysis

**Figure 9:**
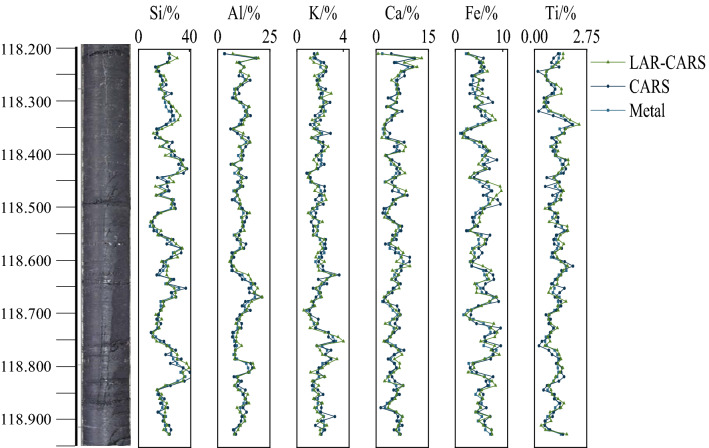
The effect of in-situ core detection.

The purpose of this study is to develop a data fusion technique that can be applied to in-situ core detection. Therefore, the CARS-XGBoost and the LAR-CARS-XGBoost were specifically applied to a fresh core detection. At the same time, this section of core was placed in ITRAX Core scanner for scanning analysis. Before scanning, the core scanner was corrected with the sample results of wavelength dispersive X-ray fluorescence spectrometer to ensure the consistency of the scanning results of the two instruments. The sampling interval of the instrument on the core was 1 cm, and other parameters were set by referring to literature to obtain oxide content results, which were converted into element content, and the detection results were shown in Fig. 9. Statistical parameters are shown in Table 3.

**Table 3:** Statistical parameters of core modeling for independent drilling.

Metal	Feature selection algorithms	*R*^2^	RMSEP (%)	RPD	Metal	Feature selection algorithms	*R*^2^	RMSEP (%)	RPD
Si	CARS	0.857	3.321	2.031	Ca	CARS	0.612	1.279	1.380
	LAR-CARS	0.851	2.662	2.392		LAR-CARS	0.724	1.158	1.533
Al	CARS	0.828	1.542	1.970	Fe	CARS	0.914	0.752	2.234
	LAR-CARS	0.798	1.355	2.080		LAR-CARS	0.931	0.688	2.426
K	CARS	0.627	0.428	1.352	Ti	CARS	0.690	0.230	1.548
	LAR-CARS	0.634	0.350	1.486		LAR-CARS	0.737	0.218	1.608

According to the detection results, in the Si model, CARS and LAR-CARS had good predictive performance. In the Al model, LAR-CARS had best predictive performance. In the K model, the prediction performance of CARS and LAR-CARS was very similar, and there was almost no difference between the two models. In the model of Ca, LAR-CARS had best prediction performance. In the model of Fe, CARS and LAR-CARS had similar prediction performance. In the model of Ti, LAR-CARS had best prediction performance.

The results of in-situ core detection show that the algorithm model of LAR-CARS was generally good. In K and Fe prediction models, CARS and LAR-CARS had similar prediction performance. The RPD of K was only 1.352–1.486. The prediction accuracy was relatively poor among all models, which may be due to the fact that there were few types of inorganic and organic matter related to K and their occurrence in China was relatively small, which was similar to the report^37^. In minerals. Si had a good prediction effect, and the RPD of the two algorithm models was 2.031 and 2.392, indicating that the screened characteristic variables had a good sensitivity to Si and a high linearity. In particular, it should be noted that Fe also has a good prediction effect, with RPD of 2.234 and 2.426, which may be because Fe oxides such as Fe_3_O_4_ and Fe_2_O_3_ have obvious characteristic peaks in the visNIR spectral region. The detection accuracy of Fe-containing minerals by visNIR spectroscopy was generally good, which was similar to that reported in Ref.^38^. The results of this study show that LAR-CARS can improve the quantitative analysis of rock forming elements by screening characteristic variables of XRF and visNIR OPA, and provide necessary technical support for digital exploration.

## Conclusion

In this paper, the combination of LAR and CARS algorithm is used to establish the wavelength variable screening algorithm, which is applied to the quantitative analysis of rock forming elements in coal seam cores. By comparing the quantitative results of coal core drilling elements with five commonly used algorithms LAR, SPA, MC-UVE, CARS and LAR-CARS, the influence of characteristic variable selection algorithm on quantitative analysis of XRF and visNIR by OPA was evaluated. The results showed that LAR-CARS screened fewer variables. After the analysis of the selected variables, the characteristic variables screened by CARS and LAR-CARS were statistically related to the measured elements. In XRF detection, the absorption enhancement effect between elements will affect the linear relationship between element content and characteristic peak intensity, so the traditional XRF internal standard method is not accurate for core detection with complex matrix effect and various elements, which affects the analysis of key areas in exploration. In visNIR spectral detection, although elements do not directly have characteristic peaks in the spectral range, organic compounds, hematite and pyrite in the core all have obvious characteristic peaks in the visNIR spectral range, and these substances have a statistical relationship with element content. Therefore, the feature variable screening algorithm will select more feature peaks, and too many feature summits will affect the stability of the model. Through the comparison of the number of characteristic variables, it is found that it is of practical significance to select the spectra using LAR first and then CARS.

In this study, two drilling three-section discontinuous cores were selected as the research object, one of which was selected as LOOCV set and the other as independent validation set. The prediction results of LOOCV set showed that the LAR-CARS had a good stability in the detection of six rock forming elements (Si, Al, K, Ca, Fe and Ti). The validation set effect showed that the models established by CARS and LAR-CARS can be well applied to the validation set. The RPD of other models (LAR, SPA, MC-UVE) was over 1.4, indicating that these models had certain predictive performance. Among them, the prediction ability of LAR was poor, which was related to the large number of feature variables screened by LAR. High dimensional matrix will reduce the generalization ability of quantitative model. Too many characteristic variables have multicollinearity, which affects the accuracy of quantification.

The ultimate goal of this study is to develop a portable technique for in-situ detection that can facilitate detailed scanning of key exploration areas. Therefore, CARS and LAR-CARS models with good performance were used to test a section of core separately, and the results were compared with the ITRAX Core scanner instrument commonly used in the core scanning industry. The results showed that RMSEP of CARS and LAR-CARS models were small, and the RPD ranged from 1.424 to 2.514. All these results showed that the high-dimensional matrix formed by XRF and visNIR integration combined with LAR-CARS can be used for quantitative analysis of rock-forming elements in in-situ coal seam cores, and the analysis results can be used as the basis for judging lithology. The research will provide necessary technical support for digital mine construction.

## Data Availability

The data used to support the findings of this study are available from the corresponding author upon request.
